# Medical needs related to the endoscopic technology and colonoscopy for colorectal cancer diagnosis

**DOI:** 10.1186/s12885-021-08190-z

**Published:** 2021-04-26

**Authors:** Juan Francisco Ortega-Morán, Águeda Azpeitia, Luisa F. Sánchez-Peralta, Luis Bote-Curiel, Blas Pagador, Virginia Cabezón, Cristina L. Saratxaga, Francisco M. Sánchez-Margallo

**Affiliations:** 1 Jesús Usón Minimally Invasive Surgery Centre, Ctra. N-521, Km 41.8, 10071 Cáceres, Spain; 2 Biobanco Vasco, Fundación Vasca de Investigaciones e Innovación Sanitaria (BIOEF), Ronda de Azkue, 1, 48902 Barakaldo, Spain; 3 TECNALIA, Basque Research and Technology Alliance (BRTA), Parque Tecnológico de Bizkaia, C/Geldo. Edificio 700, E-48160 Derio, Bizkaia Spain

**Keywords:** Medical needs, Endoscopy, Colorectal cancer, Detection, Classification, CAD software

## Abstract

**Background:**

The high incidence and mortality rate of colorectal cancer require new technologies to improve its early diagnosis. This study aims at extracting the medical needs related to the endoscopic technology and the colonoscopy procedure currently used for colorectal cancer diagnosis, essential for designing these demanded technologies.

**Methods:**

Semi-structured interviews and an online survey were used.

**Results:**

Six endoscopists were interviewed and 103 were surveyed, obtaining the demanded needs that can be divided into: a) clinical needs, for better polyp detection and classification (especially flat polyps), location, size, margins and penetration depth; b) computer-aided diagnosis (CAD) system needs, for additional visual information supporting polyp characterization and diagnosis; and c) operational/physical needs, related to limitations of image quality, colon lighting, flexibility of the endoscope tip, and even poor bowel preparation.

**Conclusions:**

This study shows some undertaken initiatives to meet the detected medical needs and challenges to be solved. The great potential of advanced optical technologies suggests their use for a better polyp detection and classification since they provide additional functional and structural information than the currently used image enhancement technologies. The inspection of remaining tissue of diminutive polyps (< 5 mm) should be addressed to reduce recurrence rates. Few progresses have been made in estimating the infiltration depth. Detection and classification methods should be combined into one CAD system, providing visual aids over polyps for detection and displaying a Kudo-based diagnosis suggestion to assist the endoscopist on real-time decision making. Estimated size and location of polyps should also be provided. Endoscopes with 360° vision are still a challenge not met by the mechanical and optical systems developed to improve the colon inspection. Patients and healthcare providers should be trained to improve the patient’s bowel preparation.

**Supplementary Information:**

The online version contains supplementary material available at 10.1186/s12885-021-08190-z.

## Background

Colorectal cancer (CRC) is the third most commonly diagnosed cancer globally, representing 10% of all cancer diagnoses. In terms of mortality, CRC is the second most deadly cancer worldwide, causing about 9.4% of deaths [1]. Adenocarcinoma accounts for 96% of all CRCs [2]. Despite this, cure rates can reach up to 90% if patients are diagnosed in early stages of the disease [3]. Consequently, this would reduce the duration and costs of diagnosis and treatment, as well as future reoperations [4].

**Fig. 1:**
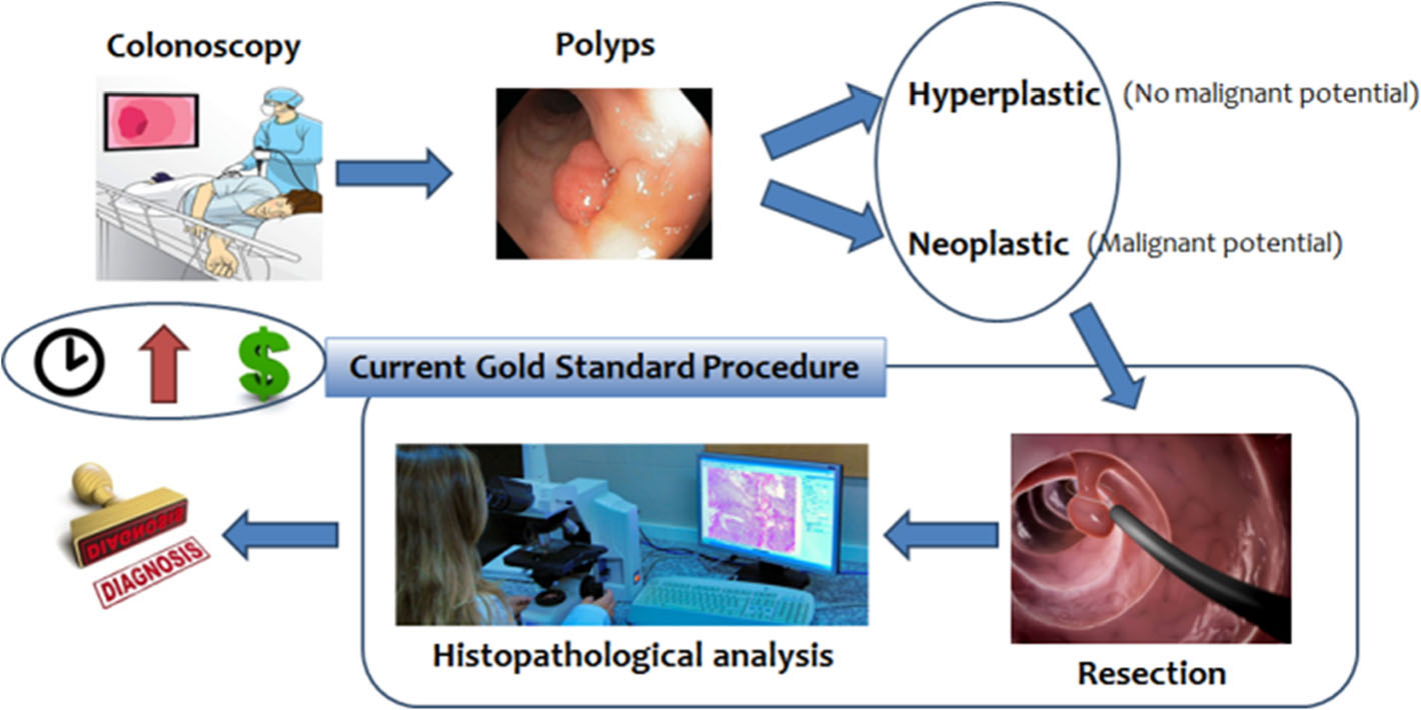
Current gold standard procedure

Screening programmes allow for early detection of CRC. Colonoscopy, which is the main detection method, reveals that up to 40% of patients have one or more polyps [5], with potentially non-malignant hyperplastic polyps being the 29–42% of them, and neoplastic ones with malignant potential the rest [5]. However, almost 30% of the polyps are not detected [6], so it is important to improve the adenoma detection rates (ADR), as every 1% of its increase is associated to a 3% decrease of CRC risk, and 5% decrease of mortality related to CRC [7]. In the current gold standard procedure (Fig*.* 1), all polyps (both hyperplastic and neoplastic) are resected and sent to histopathological analysis for diagnosis, although strategies as “diagnose and leave” for hyperplastic polyps or “resect and discard” for neoplastic polyps could be followed, depending on the used diagnostic technique and the experience of the endoscopist [8, 9]. This standard diagnostic clinical procedure still depends on biopsy, tissue sample preparation and detailed analysis by an expert pathologist, which includes extraction, preparation, cutting, and staining with Hematoxylin-Eosine to assess the morphological pattern. This protocol implies high diagnostic time and costs and may unnecessarily expose patients to the risks associated to polypectomy, besides the high psychological impact that the waiting time might cause [10]. While adenomatous polyps have malignant potential and must be resected to protect against CRC, hyperplastic polyps have not and can be left.

Recent large multi-centre studies [11, 12] have used advanced imaging modalities such as narrow band imaging (NBI) and visual classification schemes, such as NBI International Colorectal Endoscopic (NICE) or vascular pattern intensity, to differentiate hyperplastic from adenomatous polyps during colonoscopy. However, these procedures are not a reliable enough as replacement method for histological analysis, with accuracy well below the recommended levels, and unsuitable to be applied outside expert academic centres as it highly depends on the clinician’s experience and training [13, 14].

On the other hand, clinicians lack tools for the assessment of lesion margins, both prior and after resection. Incomplete polyp resection, especially when larger lesions are removed in piecemeal fashion, significantly increases the tumour recurrence probability [15] and might be the cause of post-colonoscopy CRC in up to 50% of the cases [16, 17]. Regretfully, it is currently difficult to study the progression of partially removed polyps [18].

Moreover, when a polyp invades the submucosa, it is classified as T1 CRC and since the risk of lymph node metastasis increases, surgery is recommended [19]. Although the percentage of lymph node metastasis is lower than 15% [20, 21], determining in-situ the invasion depth could allow identifying patients with higher risk of lymph node metastasis to provide them adequate treatment, as cure rates of T1 CRC patients reaches up to 85% with only endoscopic treatment [19]. However, determining invasion depth is still a challenging problem for non-invasive existing techniques such as endoluminal ultrasound [22] or NBI [23].

Therefore, improved diagnostic techniques are required to differentiate hyperplastic from neoplastic polyps, allowing in situ non-invasive assessment, safe characterization and most appropriate resection method of lesions during colonoscopy. New systems that provide accurate and objective optical identification with software support at the time of colonoscopy would reduce time and costs of diagnosis. In fact, the European Society of Gastrointestinal Endoscopy has recently identified the use of optical diagnosis as one of the key research questions that gastroenterology faces [24]. However, in order to improve the design and development of such new technologies, it is important to previously identify precise medical needs and colonoscopy procedural constraints, which is the objective of this work.

## Methods

### Design and materials

To identify medical needs, a 2-step process has been followed. Initially, a reduced number of endoscopists were interviewed to extract a draft list of needs, which was later confirmed and improved by surveying a broader number of clinicians.

#### Interviews

The semi-structured interview technique allows respondents to freely express their point of view in their own terms. It has been used in the first step of this study, as it is considered a versatile and flexible method [25]. The interviewer followed an interview guide, which included a list of questions in a predefined, preferred order that should be covered during the conversation. The interview guide included four blocks of questions (see Additional file 1), ranging from generic to concrete, which were based on literature review relevant findings. Such review was conducted with the terms *endoscopy, colonoscopy, interview, colorectal cancer* using PubMed, Google Scholar, Web of Science for studies published between 1990 and 2015. These questions were revised by independent clinicians, including gastroenterologists, endoscopists and pathologists, to assure that the final set of questions covered all relevant issues. Each interview was expected to last approximately 30 min and was carried out in the clinicians’ mother tongue. Endoscopists were the selected profile for these interviews according to their knowledge and experience. Both tape-recording and written-notes strategies were used to register these open-end questions interviews. Later, these records were transcribed and further analysed together with written notes.

#### Survey

An online questionnaire was developed using Survey Monkey [26], aiming to contrast conclusions drawn from the interviews with a broader number of clinicians. A preliminary draft questionnaire was designed based on the most relevant results from the interviews, but also supported by a review of related studies. The search strategy for such review was conducted using PubMed, Google Scholar, Web of Science for studies published between 1990 and 2015, with the terms *endoscopy, colonoscopy, questionnaire, survey, colorectal cancer*. In first place, after choosing the language (Spanish, English or German), the respondent was asked to accept an informed consent to access the questionnaire. It contained three blocks of questions: 1) demography; 2) detection of colorectal lesions; and 3) characterization of colorectal lesions. A survey pilot test was performed with a set of experts to estimate its duration, check the understandability, and detect words that might lead to confusion. The final questionnaire can be found in Additional file 2. The questionnaire was distributed via e-mail to previously identified contacts from participating institutions and through relevant scientific societies. The e-mail body text described the study objective and specified indications to fulfil the questionnaire. All answers were kept anonymous.

### Participants

#### Interviews

Four endoscopists from “Hospital San Pedro de Alcántara” in Cáceres (Spain) and two from the “Hospital de Basurto” in Bilbao (Spain) were interviewed. The theoretical saturation criterion was used to determine the final number of interviews.

#### Survey

To conduct sampling of endoscopists participating in the study, the non-probability technique called convenience sampling has been selected. In all, 133 endoscopists from 15 countries participated in the survey and 103 (75.74%) completed answers were gathered. Incomplete answers were excluded from the analysis.

### Ethical considerations

This study exempted from review by the Institutional Review board because it was not within the scope of Law 14/2007 of 3rd July on Biomedical Research. This national regulation indicates that human health-related research involving invasive procedures needs approval by the ethical committee, but in this study the participants were not involved in invasive procedures. For the same reason, it was not considered necessary for participants to sign a written informed consent. Only verbal informed consent was considered sufficient. All participants were informed about the objectives, length and procedure of the study, the institutions responsible for the research, the contact people, the confidentiality and anonymity of the research data, and that their participation was voluntary, and they could withdraw from the study at any time if they wished to.

### Analysis

#### Interviews

Topic modelling is a statistical analysis technique widely used in free text analysis that automatically identifies latent topics in a collection of documents, and derives hidden patterns exhibited by this collection. This technique is applied to any subject that contains free-text data, including interviews [27], and also in the health care area [28]. Within the possible implementations, the Latent Dirichlet Allocation (LDA) method [29] has been selected. More deeply, by using LDA we estimate the distribution of topics per document and the distribution of words per topic in a collection of documents. This latter distribution allows representing most relevant words in word cloud charts.

#### Survey

The questionnaire includes categorical questions, except for two open-ended ones, so they are described using counts and percentages. To compare independents samples, independent χ^2^ tests with Fischer correction were used. Considered study groups were: 1) experts (> 1000 colonoscopies performed) vs non experts (≤1000 colonoscopies performed), 2) qualified endoscopist with special interest in cancer screening and/or therapeutic endoscopy vs. qualified endoscopist without any special interest, and 3) experts qualified endoscopists with interest in cancer screening vs experts qualified endoscopists without any special interest. A *p* value under 0.05 was considered statistically significant. Statistical analysis was performed with IBM SPSS Version 23 statistical package.

## Results

### Demographics

#### Interviews

A total of six endoscopists from two Spanish hospitals, “San Pedro de Alcantara” in Cáceres and “Hospital de Basurto” in Bilbao, were interviewed. Five of them were male and only one female, being between 32 and 55 years old. Median age was 51.5 [95% Confidence Interval: 44.75;55] years and all of them were senior doctors with more than a thousand of colonoscopies performed.

#### Survey

In all, 120 (90.2%) endoscopists, from a total of 133 surveyed, agreed to complete the survey. However, 17 (14.2%) did not respond to any question so 103 questionnaires were finally analysed, 68.0% in Spanish, 25.2% in English and the remaining 6.8% in German. Nearly all surveyed endoscopists (95.2%) were European (namely, from Spain, Germany, Switzerland, Italy, Macedonia, Poland, Portugal, Romania, Slovenia, Sweden, United Kingdom), and only three from South America (Paraguay and Peru), one from Asia (India) and another one from Africa (Egypt). 91.2% of surveyed endoscopists were qualified and over two-thirds (72.1%) were interested in cancer screening or therapeutic endoscopy. Overall, 78.2% of participants had performed more than 1000 colonoscopies during their career.

### Global results

After analysing the survey results, no significant differences have been found in either of the three considered study groups. This analysis suggests that existing medical needs would be equally perceived by all endoscopists, and would be not biased by belonging to one group. However, this should be considered with caution as qualified endoscopists with special interest in cancer screening and/or therapeutic endoscopy and expert endoscopists are overrepresented. Therefore, only global results from interviews and surveys of all participants are discussed, from which the medical needs regarding the colonoscopy procedure and the used technology can be extracted and divided in three different fields: clinical needs, Computer Aided Diagnosis (CAD) system needs and operational/physical needs. Verbatim quotes from participants are provided in Additional file 3.

### Clinical needs

From a clinical point of view, the main concern for endoscopists is to improve polyp detection, especially for flat polyps, to reduce the polyp missing rate. Once polyps have been detected, the next identified need is to minimize the resection of hyperplastic polyps following the “diagnose and leave” strategy. Furthermore, this concern leads to the clinical need related to in-situ classification of detected polyps, so that either the “resect and discard” or “diagnose and leave” strategies can be followed if deemed necessary. This way, costs associated to extraction, processing and analysis of hyperplastic polyps could be reduced, as well as the time for diagnosis, minimizing negative psychological impact in patients, as stated by an endoscopist. Clinicians also need to reduce recurrence rate by assessing lesion margins and inspecting the remaining tissue, avoiding re-interventions which negatively affect the patient prognosis and increase the risk of complications (such as bleeding, perforation, etc.). Equally important is to assess the depth of submucosal invasion for detected lesions, as it is an essential parameter to decide whether endoscopically resect early colorectal cancerous lesions.

Survey results (Table 1) corroborate these above-mentioned needs from the interviews. Endoscopists demanded tools to improve the polyp detection. They felt that current methods for detection are insufficient and therefore that current approaches could be improved by using advanced technologies for polyp detection and characterisation. The rapid identification and classification of polyps was considered as clinically useful. In fact, 88.0% of respondents indicated that they would like to have an automated system that would suggest the diagnosis without having to make an interpretation of the lesion.

**Table 1:** Clinical Needs

	N	%
*An automated system would be preferred*	81	88.0
*For which type of lesion would a Computer Aided Diagnosis (CAD) system be MOST useful?*		
Flat/Depressed Polyps	87	89.7
Sessile Polyps	3	3.1
Pedunculated Polyps	7	7.2
*What information would you like to obtain the MOST?*		
Infiltration probability	70	83.3
Depth estimation	60	71.4
Diagnosis suggestions	55	65.5
Margin estimation	53	63.1
Grade	30	35.7

The overall preference was a tool to detect flat or depressed polyps, as 89.7% of endoscopists indicated that this assistance would be more beneficial for this type of lesions, reflecting the consensus that they are more difficult to detect than elevated lesions. The most desired characteristic to be provided by such system is the infiltration probability (83.3%), followed by the estimation of the polyp depth (71.4%), a diagnostic suggestion (65.5%) and polyp margins (63.3%). All these features would assist on deciding how to proceed and potentially avoid unnecessary biopsy or polypectomy if demonstrated to be of adequate accuracy.

### Computer aided diagnosis (CAD) system needs

According to the results from the interviews, endoscopists expect the software, or computer aided diagnosis (CAD) systems, to support the polyp detection and diagnosis with additional information for a better diagnosis. They also demand that the CAD system provide them with information about physical characteristics of the polyps, such as the size, and its location. Besides including polyp detection and characterization in the CAD system, it is also important to accurately visualize the polyp contour for safe margin resection and avoiding thus recurrence rates. Clinicians stated that a CAD system that indicates polyp invasiveness or remaining adenomatous tissue left after resection is needed, as it is currently not available. Finally, for a better understanding and a user-friendly environment, all this information provided by the CAD system is requested to be mainly visual.

Survey also shows these trends (Table 2), as most endoscopists prefer that the CAD system provides feedback as visual cues to show polyp detection directly highlighting the polyp on the endoscopic image (also called augmented display) (72.7%) instead of being shown on the screen away from it (43.4%). Regarding polyp diagnosis suggestion (classification), a high proportion of endoscopists (83.3%) preferred a ‘traffic light’ display, with different colours representing different histological predictions, rather than text (31.0%) or an audible alarm (3.6%).

**Table 2:** CAD System Needs

	N	%
*According to your experience, which is the MOST helpful indicator to establish the malignancy of a lesion?*		
Paris classification	21	23.3
Vascularity	12	12.9
Lesion surface (granularity / no granularity)	11	12.1
Kudo’s pit pattern	44	47.8
*Existing methods are NOT simple or reproducible enough*	31	34.1
*How would you prefer to be alerted to the detection of a polyp?*		
Audible alarm	11	11.1
Visual cue on screen (away from the endoscopic image)	43	43.4
Highlighting the polyp on the endoscopic image (augmented display)	72	72.7
*How would you like to receive the feedback on the diagnosis suggestion?*		
Audible alarm	3	3.6
Traffic light cues (Green – benign, Yellow – pre-malign, Red – malign)	70	83.3
Text	26	31.0
*I would completely remove large non polypoid lesions with more confidence if I had a CAD system*	79	86.8
*The CAD system could contribute to detect the residual lesion of a piecemeal polypectomy scar*	84	92.3

On the other hand, the most helpful method to establish the malignancy of polyps is the Kudo’s pit pattern (47.8%), over the Paris classification (23.3%) or the vascularity (12.9%). Therefore, future developments should consider presenting information in this manner, although more than half of the participants (65.9%) think that the existing methods to establish the malignancy of a lesion are not simple or reproducible enough.

### Operational/physical needs

Interviews with panels of expert endoscopists gave also insights into the operational needs. Firstly, the morphology of the colon itself makes it difficult to visualize and detect certain polyps. For that, they demanded better lighting inside the colon to perform the colonoscopy, shortening thus the time spent in reviewing or cleaning dark areas. To meet the need for a better visualization and exploration of the mucosa, the use of flexible/rotatory tips in the endoscope should be further explored. This way, polyps located in hidden areas of the colon could be detected by just rotating the tip. However, the flexibility of the instruments to be inserted is also a concern for endoscopists, as they should be flexible enough to pass through the working channel, but rigid enough to transmit the movements and force from outside. Finally, endoscopists would like to use equipment with better image quality.

### LDA results

The interviews to analyse consist of a collection of six documents, one for each interviewed endoscopist. After applying the LDA method, two topics that best describe this collection were found. For each topic, the words distributions were represented as word cloud charts, so the larger the occurrence of a word is, the bigger and bolder it appears in the chart. Details of the LDA results are provided in Additional file 4.

The first topic revolves around the patient (counted 22 times). This might be because some technical problems during colonoscopy are mainly related to the patient. While some can be easily overcome, such as the poor preparation of the colon before the colonoscopy, others are intrinsic to the patient, such as the anatomy of the colon in terms of folds or haustrum. In any case, they complicate to see all polyps and dark zones, as well as the progress of the endoscope. Moreover, since colonoscopy has to be performed in a limited time, these patient-related problems also reduce the exploration time. Lastly, reducing the time until the diagnosis has not much impact on the patient’s prognosis but rather an executive/management impact.

In the second topic, *polyp* stands out over the rest of words (counted 36 times), suggesting that the focus is placed on correctly knowing how to detect and identify the polyp based on endoscopy imaging, as supported by other words in the topic, such as the ones equivalent to *polyp,* like *lesion*, *injury,* or *adenomatous*, other related to placements of polyp, like *position*, *area*, or *colon*, or actions about the polyp, like *measure*, *detect*, *locate*, or *identify*.

## Discussion

The findings of this study provide information from the endoscopists’ point of view about the main needs in the context of endoscopic technology and the colonoscopy procedure currently used for the diagnosis of CRC. Results from interviews and questionnaires revealed that needs might be clustered into three different groups: (a) clinical needs, mainly related to problems in polyp detection and classification, especially flats polyps, as well as their location, size, margins and penetration depth or invasiveness more precisely during colonoscopy; (b) CAD system needs, demanding visual information/alarms for assisting on the polyp characterization and diagnosis, and (c) operational/physical needs, especially in terms of equipment limitations related to image quality and colon lighting, poor bowel preparation or the flexibility of the endoscope tip.

### Clinical needs

For better in-situ polyp detection and classification, as demanded by clinicians, various technologies are available to enhance the endoscopic image. Firstly, the NBI highlights the contrast between vascular structures and the surrounding mucosa [30], while the Flexible spectral Imaging Color Enhancement (FICE) technique, and its extension Blue Laser Imaging, consider morphological features, pit pattern and vessel characteristics of polyps [31, 32]. On a different approach, the linked color imaging finds pixel color differences in neoplastic and healthy tissue [33]. Lastly, the iScan provides real time virtual chromoendoscopy for a detailed view of the mucosal and vascular patterns [34].

Besides these techniques, the standard polyp detection with white light colonoscopy can be improved further by means of fluorescence [35, 36] or autofluorescence [31] imaging. This last approach does not use exogenous agents employing both visible [37] and near-infrared [38] light. In contrast to this, endocytoscopy is a high-resolution microendoscopy technology that detects fluorescence after the systemic or topical administration of an exogenous fluorophore [39].

The emerging advanced optical imaging technologies, such as confocal fluorescence microscopy (CFM), optical coherence tomography (OCT), Raman spectroscopy (RS), hyperspectral spectroscopy or multi-photon tomography (MPT), show great potential for assisting clinicians in the early detection of cancerous diseases [40]. CFM provides real-time microscopic images underneath the surface mucosa of 40–70 μm depth, but requires expensive equipment, significant technical expertise and exogenous fluorophore. In case of OCT, this technology provides high-resolution assessment of subsurface structures without contrast agent. However, there might be a delay in image acquisition and in vivo studies in humans are still lacking. RS details the tissue composition at a microscopic level and spectral information is suitable for automated analysis, but further characterization in humans and the development of endoscopic platforms is required. Finally, MPT high-resolution images show histologic features and changes in tissue without contrast agent, but further characterization in humans and development of endoscopic platforms are missing. Thus, combining imaging technologies that offer complementary information so flaws can be compensated is at stage on the current development of new diagnostic solutions [31].

The above-mentioned enhanced imaging techniques have been commonly applied to identify and characterize colonic lesions but they are less focused on identifying incomplete excision and residual tissue during polypectomy or recurrent disease on follow up, even though there is some evidence of its potential [41]. As for NBI, its effectiveness has not been shown to determine complete excision of diminutive polyps (< 5 mm) [42], but the detection of recurrence when examining previous scars was enhanced when combined with high definition (HD) white light [43]. On the other hand, chromo-endoscopy with 0.13% indigo carmine has showed a significant increase in residual polyp detection compared to alone while light endoscopy in predominantly larger polyps (> 5 mm) [44]. Alternatively, the Fourier transform infrared spectroscopy, that provides chemical analysis at the molecular level, helps determining whether the resection margin contains foci with recurrence potential [45].

Finally, regarding the clinical need to estimate the depth of submucosal infiltration of polyps, the endoscopic ultrasonography (EUS) and narrow band imaging-magnifying endoscopy (NBI-ME) are often used. The axial images of the tumors produced by EUS could detect the invasion of the normal layered structure of the colorectal wall [46], while the NBI-ME combination could provide visualization of capillary vessels and their fine structure in the surface layer [47, 48]. A comparative study has shown that NBI-ME is more appropriate for estimating the early CRC invasion depth before treatment to avoid unnecessary surgery, whereas EUS should be applied to early CRC in which the decision to conduct endoscopic resection is difficult [49]. Furthermore, the probe-based confocal laser endo-microscopy is also useful for the differentiation of normal submucosa from carcinoma infiltration, especially when is accompanied by severe fibrosis. This technique provides more sensitivity and accuracy than magnifying chromo-endoscopy, but large-scale prospective studies are needed to further evaluate the clinical impact of its use during endoscopy [50].

### CAD system needs

The imaging technologies should be complemented with advanced image processing methods that facilitate the detection, analysis and diagnosis of polyps on real time through a CAD system.

On the one hand, many detection methods based on artificial intelligence are already available, although clinical evaluation is still missing [51]. ENDOANGEL helps improving the ADR by identifying endoscope slipping and previously seen frames, thus avoiding blind spots [52]. Similarly, the Automated Polyp Detection Software (APDS) [53] analyses colours, structure, texture and motion information of polyps, and marks a region of interest with small green rings on the endoscopic image. This visualization method matches the preferred option in this study. However, APDS should be further improved before its actual clinical application as it did not detect more polyps than those already detected by clinicians [54]. Wang et al. [55] present an automated polyp detection system which was used during colonoscopy providing as output simultaneous visual and sound alarms. This system increased the ADR, predominantly thanks to the detection of hyperplastic polyps and diminutive adenomas, but there was no difference in the detection of advanced adenoma or sessile serrated lesions [53]. Finally, the GI-Genius system shows a faster reaction time for polyp detection than endoscopists while increasing also the ADR [56]. Nevertheless, there is still room for improvement and methods based on deep learning and/or machine learning are still being developed to explore different approaches, such as the extraction of fractal dimension of wireless capsule endoscopy images used for the identification of abnormal frames [57], or the use of a weakly supervised convolutional neural network that can be trained solely with semantically annotated images, indicating whether they contain anomalies or not [58].

Efforts have also been focused on CAD systems for the classification and diagnosis of colonic polyps. EC-CAD performs an analysis of nuclei and texture from endocytoscopic images of colonic tissue to estimate the probability of non-neoplasm, adenoma and invasive cancer [59] and on the same line, but using endocytoscopy with NBI, a CAD system evaluates cellular, glandular, and vessel structures of colonic polyps and provide the probability of neoplastic or non-neoplastic diagnosis [60]. Similarly, an artificial intelligence model for real-time characterization of colorectal polyps has been developed based on NBI video frames [61]. This system grades the polyp according to the NICE classification into type 1 – hyperplastic or type 2 – adenomatous and provides the associated probability and a credibility score indicating the confidence in the diagnosis.

The estimation of the polyp size influences the diagnosis treatment, so clinicians demands it to be automatically provided by the CAD system, as the polyp size determination through open forceps is subject to error [62]. The challenge is to recover the spatial information for size estimation of a 3D polyp from a colonoscopic 2D image. So far, little progress has been achieved in this regard. A method has been developed for the polyp size classification from colonoscopic videos into under and over 10 mm [63]. Just like the size, the location of the polyp also influences the risk of CRC [64], so it is also interesting to estimate the location of polyps (in mm from the anus), and not only indicate segment they are in.

### Operational/physical needs

Besides addressing the above-mentioned clinical and CAD system needs, new imaging technologies with high resolution could also help to reduce the problems of colon lighting for better polyp detection as requested operational/physical needs. CAD systems are mainly focused on the detection of polyps within the endoscopic field of view [54] so their performance is highly dependent on the proper exploration of the colonic mucosa and polyp exposure, as 20% of the colon surface is never surveyed [65]. To solve this limitation, Stanek et al. [66] have developed a CAD system to identify dark or hidden areas of the colon and alert the endoscopist, so that they can be explored more thoroughly. This software assesses the video frame quality and withdrawal spiral motion of the colonoscope to display a green marker in real-time when a quadrant of the image is inspected. Other approach is focused on the development of a 3D map of the colon by predicting the depth from endoscopic images, so the localization of the lumen is used to assist navigation during colonoscopy [67], thus providing a real-time quantitative measure of the colon inspection.

However, besides lighting of the colon, the poor preparation of patients’ bowel prior to colonoscopy highly influences polyp detection. Although new trends of split-dose regimen of bowel preparation has been associated with a better colonic cleansing and adenoma detection, the reticent patient attitude towards this split regimen is still a barrier [68].

To maximize the colon exposure, mechanical add-on devices are also used [54], such as a transparent hood, balloon colonoscopy, or wide-angle colonoscopy [69]. These devices have greatly improved polyp detection rates by expanding the visual field behind the folds. Other accessory device-based systems, such as the Endocuff Vision or EndoRings, manipulate the colon folds using radially extended flexible projections [70, 71]. A cheaper and easy-to-access alternative could be the use of a 3D-printed cap adjustable to standard endoscopes with sideoptics [72], which incorporates two micro-cameras fixed to achieve additional views. Approaches like these could meet the operational/physical limitations of current colonoscopes in terms of flexibility or tip stiffness that makes it difficult to find polyps (especially flat ones) located behind the folds or near the colonic flexures.

Availability of endoscopic systems with better image quality is also requested. Current colonoscopes are mainly based on HD images, but sometimes with this resolution images are still blurred, and it is difficult to distinguish the different tissues. Nowadays, some deep learning algorithms are been implemented for imaging improvement, however they can only process 3–6 frames per second (fps) on 4 K videos and 2fps on 8 K videos, far from real-time requirement (25–30 fps) for endoscopy applications [73].

### Challenges

According to the discussion, different challenges still need to be addressed to meet the identified needs. Regarding clinical needs, the assessment of lesion margins and inspection of the remaining tissue of diminutive polyps with less than 5 mm with imaging techniques should be further improved, with the aim of reducing recurrence rates, since in this kind of polyps it can reach up to 11% [45].

In case of CAD system needs, additional efforts are encouraged in the development of CAD systems for the detection of flat polyps, as they are problematic for endoscopists and have a considerable prevalence of almost 25% [74]. Moreover, a CAD system that estimates the polyp size in real time, rather than classifying it into categories, should be developed.

The first challenge related to operational needs would be the development of a tip with 360° vision, either with a rotating tip or with a system that would allow peripheral as well as focused vision. As far as we know, new scope modalities have achieved a vision of 330°, such as the Full Spectrum Endoscopy colonoscope or the Third-Eye Retroscope and Third-Eye Panoramic devices [75]. Additionally, the implementation of an accelerator algorithm for 4 K and 8 K videos appropriate for real-time detection should be further addressed.

### Future scopes

The combination of OCT and MPT photonics technologies is also a promising approach that can offer high sensitivity and specificity for diagnosis [76], representing an unprecedented powerful clinical tool to be used for both CRC early diagnosis and follow-up. These images modalities provide microscopic structural and functional information, which are not provided by NBI, FICE or similar imaging modalities.

Beyond the advances in the development of CAD systems for either polyp detection or classification, it would be desirable to incorporate both detection and classification functionalities into one single system to complement the normal workflow of endoscopists [61]. As preferred by endoscopists, visual cues should be overlaid onto the clinical display as augmented reality in case of detection, and with a “traffic light” display with different colours for the different histological classifications, based on Kudo’s pit pattern. In any case, the final decision on the diagnosis should be made by the clinician, with the CAD software acting as support tool. The opinion of endoscopists indicating that these classification methods (Kudo, Paris, etc.) are not simple and reproducible enough, together with findings from other studies showing that their interobserver variability should be improved [77], make their diagnostic predictions not fully reliable.

On the other hand, educational initiatives for patients to improve their compliance of bowel preparation are encouraged. But due to the foreseeable lack of patient involvement, such educational initiatives should also be addressed to healthcare providers. There are some independent predictive factors of an inadequate bowel preparation, such as diabetes, psychiatric illness, opioid use, active tobacco use, history of inadequate bowel preparation, and Medicaid coverage [78]. Therefore, if healthcare providers or endoscopists previously know the patients with these characteristics, they could foresee it and reinforce their education for a proper bowel preparation.

Regarding operation needs, it has been shown that 4 K ultra HD monitors reduce operative time and intraoperative blood loss in colorectal laparoscopic surgery, so these technologies with higher quality should be widely incorporated in clinical settings for the normal colonoscopy practices, since currently these 4 K ultra HD technologies are mainly available in specialized, high-volume laparoscopic centers [79]. However, the reduction of operative time achieved with these technologies should be taken with caution, as acting quickly with a shorter withdrawal time is also associated with higher interval CRC rates [80].

## Conclusions

Findings of this study provide the scientific community and clinicians with knowledge about the problems and needs that currently exist in the standard procedure of colonoscopy and the endoscopic technology used for CRC detection and diagnosis, specially focused on clinical, CAD software and physical/operational needs. Although some initiatives already address such needs, there are still some challenges to be solved.

For polyp detection and classification, the potential of optical technologies, such as OCT and MPT, should be further exploited to provide structural and functional information of colonic tissue and complement current imaging technologies. Similarly, inspection of the remaining tissue of diminutive polyps (< 5 mm) should be further improved to reduce their recurrence rates. Finally, in terms of clinical needs, further studies are needed to analyse the clinical impact of using EUS or NBI for the estimation of the depth of submucosal infiltration during colonoscopy.

To support such imaging technologies, polyp detection and classification methods based on artificial intelligence should be merged in a single CAD system to complement the regular workflow of endoscopists. Such CAD systems should automatically display visual aids for polyp delimitation, Kudo-based diagnosis as well as other relevant metadata, such as the estimated polyp size in mm, the distance from the anus or depth estimation, with the final aim of real-time classification of neoplastic and hyperplastic polyp for choosing the best approach.

Certain operational/physical limitations make it difficult to detect polyps, so further research to develop a tip with 360° vision is highly desirable alongside with wider use of equipment with better image quality than current HD systems and development of real-time processing for polyp detection. Lastly, thoughtful educational proposals for patients and healthcare providers should be implemented to improve the compliance of bowel preparation prior to colonoscopy.

## Supplementary Information

**Additional file 1.**

**Additional file 2.**

**Additional file 3.**

**Additional file 4.**

## Data Availability

The datasets used and/or analysed during the current study are available from the corresponding author on reasonable request.

## Abbreviations

CAD: Computer-aided diagnosis
CRC: Colorectal cancer
ADR: Adenoma detection rate
NBI: Narrow band imaging
NICE: NBI international colorectal endoscopic
LDA: Latent dirichlet allocation
FICE: Flexible spectral imaging color enhancement
CFM: Confocal fluorescence microscopy
OCT: Optical coherence tomography
RS: Raman spectroscopy
MPT: Multi-photon tomography
HD: High definition
EUS: Endoscopic ultrasonography
NBI-ME: Narrow band imaging-magnifying endoscopy
APDS: Automated polyp detection software
fps: Frames per second
