# Supplementary material for: Medical needs related to the endoscopic technology and colonoscopy for colorectal cancer diagnosis
Source: BMC Cancer. 2021 Apr 26;21:467. doi: 10.1186/s12885-021-08190-z (PMC8077886; doi:10.1186/s12885-021-08190-z)
Supplement: Supplementary file 1 — Additional file 1. [file 12885_2021_8190_MOESM1_ESM.docx]

***Additional File 1***

- **Block 1. Presentation (1-2 minutes)**

1. Self-introduction of the interviewer

2. Presentation of the project

3. Objective of the study

4. Outline of the interview:

- Block 2: demographic data

- Block 3: current procedure and technologies for colonoscopy and colorectal cancer diagnosis

- Block 4: challenges to be addressed to improve the current procedure and technologies

5. Acceptance to voluntarily participate in the interview

6. Permission for recording

- **Block 2. Demography (up to 5 minutes)**

1. Please, make a briefly introduce yourself: name, age, academic training, number of colonoscopies performed, where you are working or have worked, in what position, etc.

- **Block 3. Current procedure for colonoscopy and colorectal cancer diagnosis (10 minutes)**

1. What is the current screening procedure for the diagnosis of colorectal cancer?

2. But more specifically, and more related to your field of work, how is the current colonoscopy procedure?

3. What problems do you encounter while performing such procedure? And related to the technology used?

- **Block 4. Challenges (15 minutes)**

1. What improvements would you like to see in the colonoscopy procedure? And related to current technology and equipment (expected innovations)?

2. What is your opinion and personal experience of the use of advanced imaging techniques (including dye-spray, virtual chromoendoscopy, photonics techniques) for the diagnosis of polyps and colorectal cancer?

3. If you could have at your disposal an ideal device for supporting your decision-making in the assessment of polyps or colorectal cancer, what additional information would you like to be provided with? (Detection, classification, visual information, auditory information…)
