# Supplementary material for: Medical needs related to the endoscopic technology and colonoscopy for colorectal cancer diagnosis
Source: BMC Cancer. 2021 Apr 26;21:467. doi: 10.1186/s12885-021-08190-z (PMC8077886; doi:10.1186/s12885-021-08190-z)
Supplement: Supplementary file 2 — Additional file 2. [file 12885_2021_8190_MOESM2_ESM.docx]

***Additional File 2***

- **Block 1. Demographics**

1. In what country do you work?

2. Please indicate your area of practice.

- Trainee colonoscopist
- Qualified colonoscopist
- Qualified colonoscopist with a special interest in cancer screening
- Qualified colonoscopist with a special interest in therapeutic endoscopy

3. During your professional career, how many colonoscopies have you performed?

- Less than 50 procedures
- 50-250 procedures
- 250-1000 procedures
- More than 1000 procedures
- **Block 2. Detection of colorectal lesions**

4. If a software program were available to help you detecting polyps, for which type would you find it more useful? Please order from more to less usefulness according to Paris classification (1 - more usefulness; 5 - less usefulness)

- 0-Ip - Pedunculated
- 0-Is - Sessile
- 0-IIa - Flat elevated
- 0-IIb - Completely flat
- 0-IIc - Slightly depressed

5. If you had access to computer assisted diagnostic (CAD) programs, how would you prefer to be alerted to the detection of a polyp?

- Audible alarm
- Visual cue on screen (away from the endoscopic image)
- Highlighting the polyp on the endoscopic image (augmented display)
- Other (please specify)

6. If you have further comments or insights about a CAD system for polyp detection, please indicate them below.

- **Block 3. Characterisation of colorectal lesions**

7. According to your experience, what is more helpful to establish the malignancy of a lesion? Please order from more to less helpfulness.

- Paris classification
- Vascularity
- Lession surface (granularity / no granularity)
- Kudo's pit pattern
- Other

8. If you have chosen "other" in the previous question, please explain here in what you base the malignancy of the lesion.

9. Do you think the existing methods to establish the malignancy of a lesion are simple and reproducible enough?

- Yes
- No

10. Would you like to have an automated system that would give you a diagnosis, without having to make an interpretation, of the lesion you are facing (neoplastic, non-neoplastic, submucosal invasion)?

- Yes
- No

11. How would you like to receive the feedback on the diagnosis suggestion?

- Audible alarm
- Traffic light cues (Green – benign, Yellow – pre-malign, Red – malign)
- Text
- Other (please specify)

12. What information would you like to obtain?

- Diagnosis suggestions
- Grade
- Width
- Depth estimation
- Margin estimation
- Infiltration probability
- Other (please specify)

13. In the presence of large lesions that are not polypoid or with margins that are difficult to differentiate, could you completely remove the lesion with more confidence if you had a CAD system? (compared to what you are using now)

- Yes
- No

14. In the surveillance of the scar after having performed a piecemeal polypectomy, you look for residual neoplasia. Do you think that the CAD system could contribute to detect the residual lesion?

- Yes
- No

15. If you have further comments or insights about a CAD system for polyp diagnosis, please indicate them below.
