# Supplementary material for: Medical needs related to the endoscopic technology and colonoscopy for colorectal cancer diagnosis
Source: BMC Cancer. 2021 Apr 26;21:467. doi: 10.1186/s12885-021-08190-z (PMC8077886; doi:10.1186/s12885-021-08190-z)
Supplement: Supplementary file 3 — Additional file 3. [file 12885_2021_8190_MOESM3_ESM.docx]

***Additional File 3***

| **Need** | **Most representative quotes from interviews** |
| --- | --- |
| *Clinical needs* | |
| To improve polyp detection, especially for flat polyps, to reduce the polyp missing rate | *“A large polyp, larger than 1 cm, is difficult for any endoscopist to miss. The polyps that are like a mushroom, you will be able to see them easily. The problem is with those that are flat, that is, it is a roughness in the area you are seeing. So that, if you do not see it clearly or there is not a system that allows you to locate it or be able to define it well, those are the ones that are going to give you more problems.”* |
|  | *“There are different questions. The first one is to see them [all polyps] and not leave anything, and the other one is to take an attitude to a lesion that you already know.”* |
| To minimize the resection of hyperplastic polyps | *“Another limitation is having to remove all the polyps, being some polyps more dangerous and others that are not at all. This limitation forces us to remove the polyps with the risk for the patient and the expense of time and material that causes.”* |
| In-situ classification of detected polyps to reduce costs and time | *“The shorter the waiting [for histology] is, subjectively, the better, you close an episode earlier, the patient is discharged.”* |
| To reduce recurrence rate by assessing lesion margins and inspecting the remaining tissue | *“To be able to identify on a resection bed if any adenomatous residue remains. That is important.”* |
| To assess the depth of submucosal infiltration for detected lesions | *“In the case of large polyps, these usually generate major doubts when you have to say whether there is invasion or not. And also, how deep the invasion reaches, because it is not the same reaching a part of the submucosa that can be completely removed with dissection or deeper. We are talking about microns.”* |
|  | *“It is important to decide not touching this polyp because here there is a submucosal invasion, so the polypectomy to be done is going to take a risk and it will not cure the patient.”* |
| *Computer Aided Diagnosis (CAD) system needs* | |
| to support the polyp detection and diagnosis with additional information for a better diagnosis | *“I think it is also very difficult to interpret the images. We do not have a histological vision, like pathologists.”* |
|  | *“The CAD system detects the polyp and tells you that it is a polyp and draws a square, and tells you that this polyp is adenomatous, or this polyp has an area that looks like a tumor, or this polyp is hyperplastic and you do not have to do anything with it because of the place where it is located. All those things, or that it locates it with more precision.”* |
| To provide information about physical characteristics of the polyps | *“The size [of polyps], because the measurements that are made now are estimates.”* |
|  | *“A system that tells you there is a thing... it measures X, and this is hyperplastic. And then I can say in the report, at such distance there is a lesion of X millimeters, which is of hyperplastic characteristics.”* |
| To accurately visualize the polyp contour | *“I think highlighting the polyp from the rest of the mucosa would be important because sometimes you do not see very well where the edge of the polyp is. Then, if it could be delimited better by image, this would help at the time of doing the resection.”* |
| To indicate polyp invasiveness or remaining adenomatous tissue left after resection | *“Of course, no manufacturer has a system that can indicate if what I see infiltrates submucosal and if you have left an adenomatous residue.”* |
| To provide information in a visual manner | *“[That the system provides] visual [information] will always be better.”* |
| *Operational/physical needs* | |
| Better lighting inside the colon | *“The colonoscopy itself already has its difficulties: visualizing between the folds, the angulations; there are essentially flat lesions or with morphology or appearance similar to the normal mucosa, which can often be missed, and which would be desirable to identify in greater numbers, more easily. On a day-to-day basis, the tests have limited time. Many times, you spend time in washing, in cleaning properly, and the time of exploration is reduced, and it would be a good thing [to minimize any delay due to this].”* |
|  | *“Another issue is the visualization of certain areas that are slightly dark. The colon has haustra, so you look straight ahead, but there are things that you eventually might not see. Those in front, you can see them, but those that are a little aside maybe you do not see them totally or with total clarity.”* |
|  | *“Both the image capture and the lighting inside the colon. In those two fields, I think that we can move forward.”* |
| Better visualization and exploration of the mucosa | *“If you can rotate the tip more in polyps that are more on the back side of the haustrum, you could work better. Now you do not have much rotation, the tip is not so flexible.”* |
| Compromise between flexibility and stiffness of instruments | *“It is complicated because in addition the material that you have to pass through the working channel is semi-rigid. It has to have functionality from the outside. It is complicated. But that [being able to pass easily] would help.”* |
|  | *“The problem is that if you have one [endoscope] with a lot of flexibility, you do not have enough rigidity to be able to transmit the movements to the tip in an area that you have focused. For example, when you access the colon with the gastro[scope] and you have to do many laps, eventually you do not have enough stiffness to be able to reach and transmit that force.”* |
|  | *“More flexible tips are being tried, variable stiffness. But I do not see much advantage to perform the colonoscopy better with variable stiffness. That is, they are devices with which you can reach your goal in more than a 95%, but depending on the patient's anatomy, it may take more or less effort”.* |
| Equipment with better image quality | *“We all have 4K televisions at home, and here we are getting to high definition. So, I suppose it will be by miniaturization of the chips or whatever, but the imaging technology is much more advanced in household equipment, and here it has not arrived, I do not know why. I think that the more definition you have, the more you will get.”* |
|  | *“The problems of image quality, because we have mixed technology in terms of image qualities, we have high definition with no really high definition, and that is always a problem, which can sometimes be solved. You have technology, models of endoscopes with different levels of quality and image”.* |

Table 1. Verbatim quotes from participants in the interviews
