# Supplementary material for: Medical needs related to the endoscopic technology and colonoscopy for colorectal cancer diagnosis
Source: BMC Cancer. 2021 Apr 26;21:467. doi: 10.1186/s12885-021-08190-z (PMC8077886; doi:10.1186/s12885-021-08190-z)
Supplement: Supplementary file 4 — Additional file 4. [file 12885_2021_8190_MOESM4_ESM.docx]

***Additional File 4***


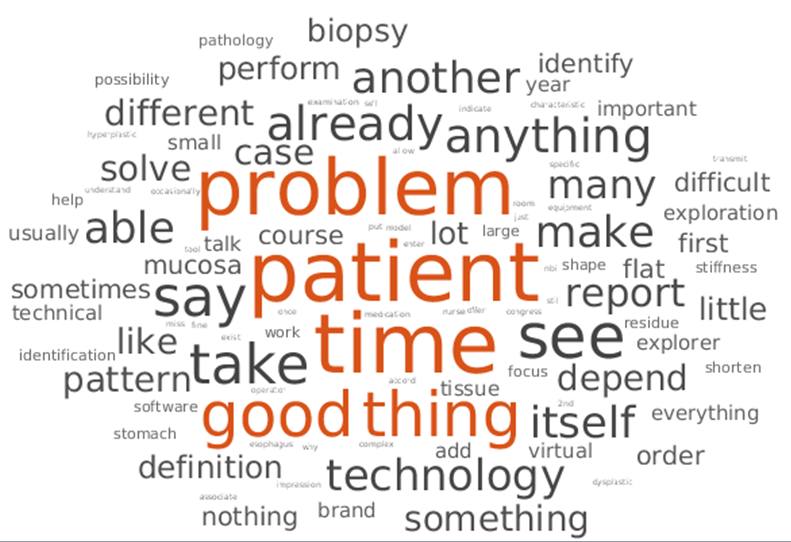


Figure 1. Words of the Topic 1, which revolves around the *patient*


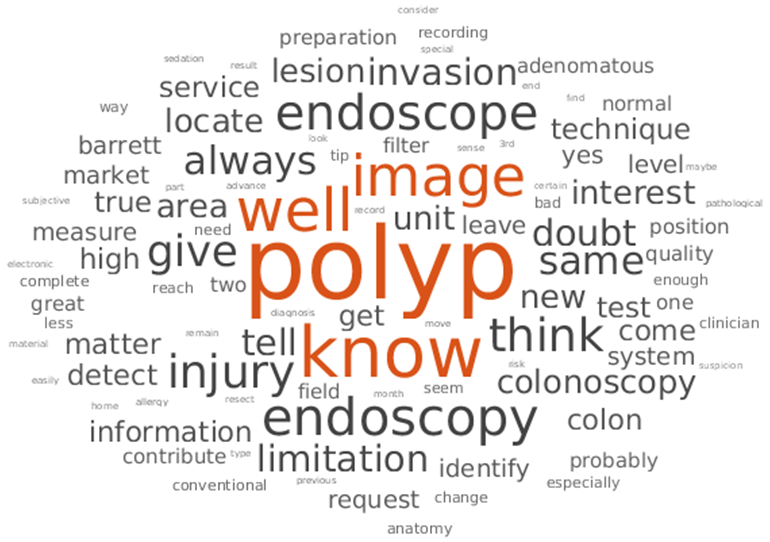


Figure 2. Words of the Topic 2, which revolves around the *polyp*


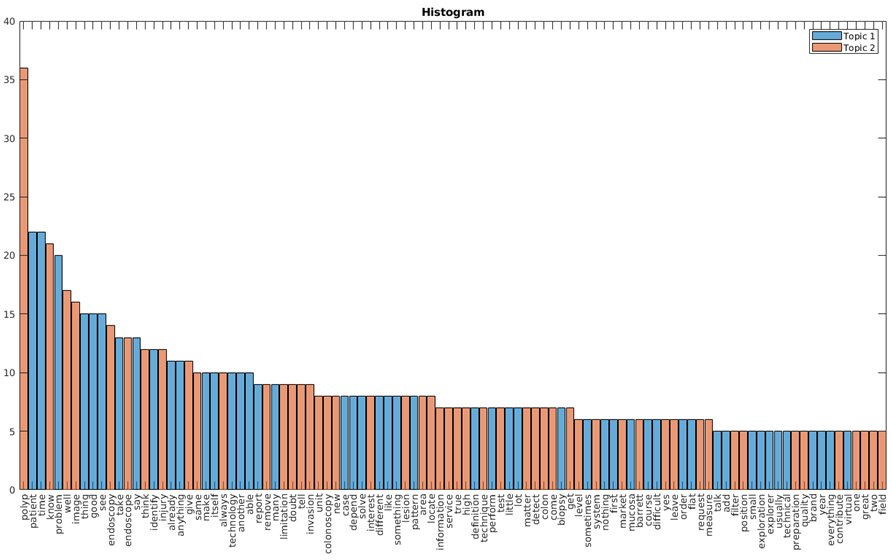


Figure 3. Histogram of the 100 words that appear the most in the interviews
